# A Modified Ridge-Splitting Technique to Restore a Completely Edentulous Maxillary Arch With a Cement-Retained Implant Prosthesis

**DOI:** 10.7759/cureus.45299

**Published:** 2023-09-15

**Authors:** Mohamed M Rahhal, Rawda Awad, Ahmed Fayyad, Hamid Nurrohman, Carlos A Jurado

**Affiliations:** 1 Restorative Dentistry, A.T. Still University, Kirksville, USA; 2 Prosthodontics, Fayoum University, Fayoum, EGY; 3 Prosthodontics, Cairo University, Cairo, EGY; 4 Restorative Dentistry & Prosthodontics, University of Texas School of Dentistry, Houston, USA; 5 Prosthodontics, University of Iowa College of Dentistry and Dental Clinic, Iowa City, USA

**Keywords:** ridge splitting, osseointegration, bone expansion, soft bone, dental implants

## Abstract

The present report describes a technique in which the maxillary bone was molded to the desired location using a series of instruments for ridge-splitting procedures. This technique aims to improve bone quality all around the implants at both the crest and apex locations. In some clinical scenarios, insufficient horizontal bone with less than 3 mm prevents implant placement. Thus, ridge splitting is a treatment of choice, and this technique creates bone expansion to form a better receptor site for endosteal implants. A case report is presented involving a completely edentulous 52-year-old male patient presented to the clinic with a horizontal bone defect. The patient complained of having difficulty eating and wants to improve his smile.

In this clinical case, a modified ridge-splitting technique was employed, differing from the conventional procedure that uses mallets, chisels, or osteotomes. A lancet and spatula were used for precise ridge splitting, followed by the placement of four endosseous tapered implants-two on each side (Dentis USA, La Palma, USA). Each implant had a diameter of 3.7 mm and a length of 10 mm. These implants were clinically placed in a single visit, with a torque of 30 N/cm² applied to ensure secure fixation. To accommodate the patient's unique maxillary bone anatomy, 25-degree angulated abutments were chosen for the four implants, ensuring a common path of insertion, and optimal angulation for long-term stability and aesthetics. Subsequently, a cemented provisional dental prosthesis restoration was fitted, and the patient reported satisfaction with both function and aesthetics.

After a period of five months of osseointegration, the stability of the implants was assessed using a resonance frequency analyzer, yielding positive results. The average resonance frequency values for the maxillary left (canine and premolar) were ISQ 68 and ISQ 71, respectively, while for the maxillary right (lateral incisor and premolar), the values were ISQ 69 and ISQ 73. These readings indicate satisfactory implant stability following the osseointegration process. The postoperative cone-beam computed tomography (CBCT) showed gain to the bone width besides better function and good results concerning the esthetics.

This report describes a modified ridge-splitting technique with a predictable and satisfactory outcome that fulfilled the patient's demands. The presented approach overcomes the disadvantages of two-staged implant placement bone grafting procedures and is also a more affordable option for the patient. CBCT evaluation confirmed bone gain with minimal morbidity after the procedure.

## Introduction

Different techniques have been proposed to reconstruct deficient alveolar ridges and to facilitate dental implant placement. These techniques typically include autogenous bone grafting, distraction osteogenesis, guided bone regeneration (GBR), and bone splitting. However, each technique has its limitations. Autogenous bone grafting, though effective, is limited by the availability of graft material and requires an additional surgical site for harvesting bone, leading to potential complications like infection and graft resorption. Distraction osteogenesis, while promoting bone regeneration, demands a lengthy treatment period and involves a complex surgical technique, often accompanied by discomfort and device-related issues due to the use of a distraction device. Guided bone regeneration (GBR) may face challenges with membrane exposure or displacement and interference from soft tissues, affecting bone regeneration. Bone splitting (ridge splitting) carries risks of bone fractures and limited width expansion for implant placement in certain cases, necessitating additional procedures. It also requires skilled practitioners due to its technical complexity. Despite these limitations, these techniques can be effective when appropriately applied, considering patient-specific factors and the expertise of dental professionals [1-3].

When teeth are lost, the amount of bone volume that is available for dental implants is typically decreased. However, it is widely believed that implant insertion must be guided by prosthetics rather than bone. Preplanned implant positioning and correct angulation are the main tools for successful implant-supported restoration [4].

A good method of bone manipulation is to do ridge splitting followed by bone expansion where a receptor site for an implant is created without attempting to remove any bone from the implant location. Maxillary bone has an inherent flexibility that can be molded to the desired location using a series of instruments. This further improves the quality of bone all around the implant, at both the crest and apex [5].

When the buccolingual bone width is 3-6 mm, ridge splitting, and bone expansion, the technique is a viable option for implant placement. The 3 mm of bone should include at least 1 mm of trabecular bone interposed between the cortical plates. This guarantees 1.5 mm of bone (cortical and cancellous) on both sides of the split ridge, allowing the bone to spread and maintain a healthy blood supply [6]. Various procedures, including split crest osteotomy, have been introduced during the last several decades. However, in situations where the buccolingual bone width is less than 3 mm, the question arises if this technique still is a viable option for implant placement [6-7].

The aim of this report is to describe the modified ridge-splitting technique bone expansion osteotomy at a width less than 3 mm done using a lancet followed by a spatula and present a case scenario with a successful outcome and time period of follow-up of 3 years.

## Case presentation

The patient is a 52-year-old male who presented to the clinic with a complete edentulous condition for upper and lower arches. The patient was wearing a mandibular complete denture. The patient's medical history evaluation revealed no personal or family history of significant medical conditions or systemic issues. Additionally, there were no reported instances of ongoing medication use that might impact the treatment plan. Clinically, the patient exhibited a narrow ridge throughout the entire maxillary arch, warranting a thorough evaluation of bone augmentation techniques to support full arch dental implant rehabilitation. His chief complaint stated the following: “I want to eat well, and I do not want a removable appliance because I want a good appearance.” The patient expressed a preference for a fixed prosthesis with the best functional outcome and the least possible cost.

After clinical and radiographic evaluation, the patient was informed of his case and the available prosthetic options, and it was agreed by both the patient and the prosthodontist to opt for an implant-supported prosthesis.

The treatment plan involved placing a total of four dental implants in the maxillary arch, with two implants on each side. Specifically, two implants were planned in the maxillary left region, addressing the canine and premolar areas. Additionally, two implants were planned in the maxillary right region, targeting the lateral incisor and premolar areas. These implant positions were carefully selected, considering the available bone and the patient's affordability, ensuring both aesthetic and functional satisfaction. Furthermore, bone grafting augmentation procedures were possibly suggested if needed to enhance the quantity and quality of the bone in the region. This step was crucial for establishing a stable foundation for the dental implants, promoting successful integration and long-term stability. Regular follow-up visits were necessary to monitor the patient's oral hygiene practices and the overall success of the implants. These visits were vital for ensuring the implants' longevity and optimal function while also addressing any potential issues promptly.

Radiographic investigation

**Figure 1:**
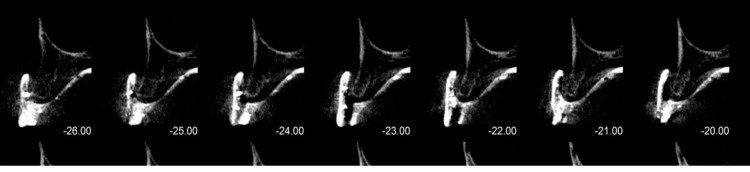
Cone Beam Computed Tomography showing bone width preoperatively at different sections

A cone beam computed tomography (CBCT) was taken to evaluate the bone quantity and quality assessment. The CBCT findings revealed poor bone quality (D3) and quantity as shown in Figure 1.

Surgical procedures

In this case, preliminary impressions were taken, and the dental casts were mounted on the articulator, allowing the prosthodontist to assess the available interocclusal space. Sufficient interocclusal space was available for accommodating the implant, abutment, and final prosthesis fixed restoration while maintaining proper occlusal relationships with the opposing dentition. Adequate space ensured that the implant-supported restoration fit comfortably within the patient's bite, allowing for proper occlusion and function during chewing and speaking.

The surgical procedures were carried out at Cairo University (Cairo, Egypt) at the postgraduate Clinic placing two implants (Dentis USA, La Palma, USA) (implant 3.7 mm diameter and 10 mm length) on each side in one visit. Local anesthesia was administered to the patients using 4% articaine (3M, St. Paul, USA) in combination with epinephrine (adrenaline) at a concentration of 1:100,000 reducing bleeding and enhancing the duration of anesthesia. This combination provided effective local anesthesia for the patients, allowing for a comfortable and pain-free dental implant surgery experience. The surgery was initiated with a maxillary crestal incision on the ridge in the anterior zone, and two vertical incisions were extended buccally.

The mucoperiosteal flap was elevated using a curette elevator (Hu-Friedy, Chicago, USA). At this point, the width of the alveolar ridge was re-examined using calibrated periodontal probes to measure the width of the alveolar ridge at the potential implant site. The measurement was typically taken at various points along the ridge to assess any variations in width. Combining the clinical examination with CBCT that was previously taken ensured a thorough and precise assessment of the narrow bone width which mandated the ridge-splitting procedure.

**Figure 2:**
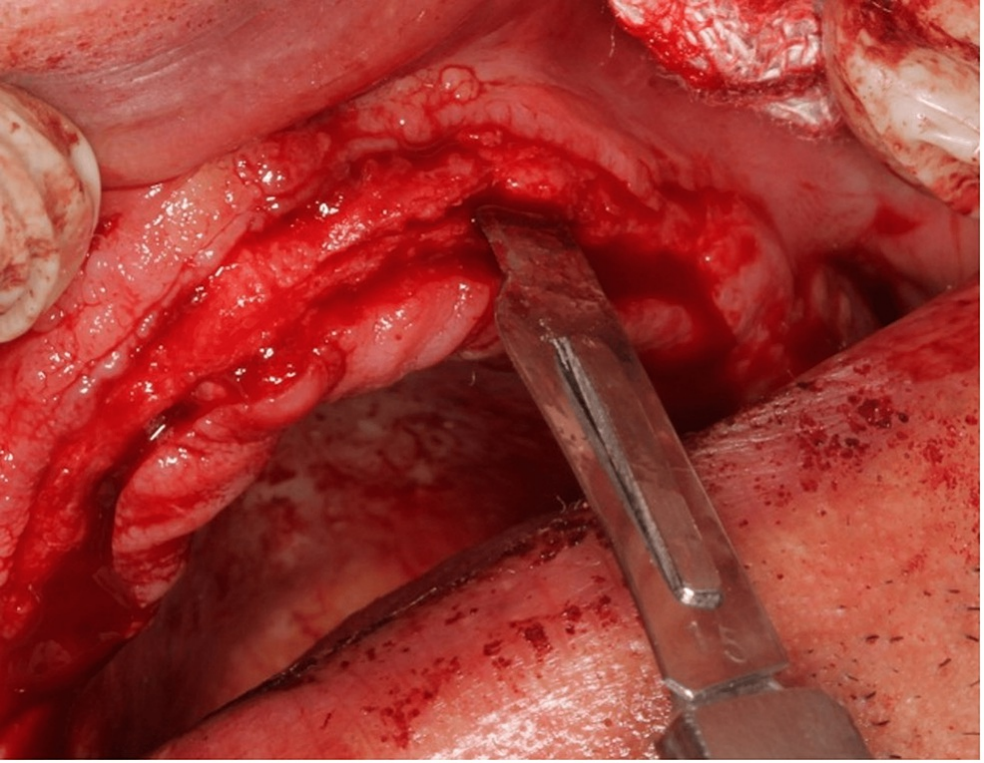
Ridge splitting initiated by using a lancet

**Figure 3:**
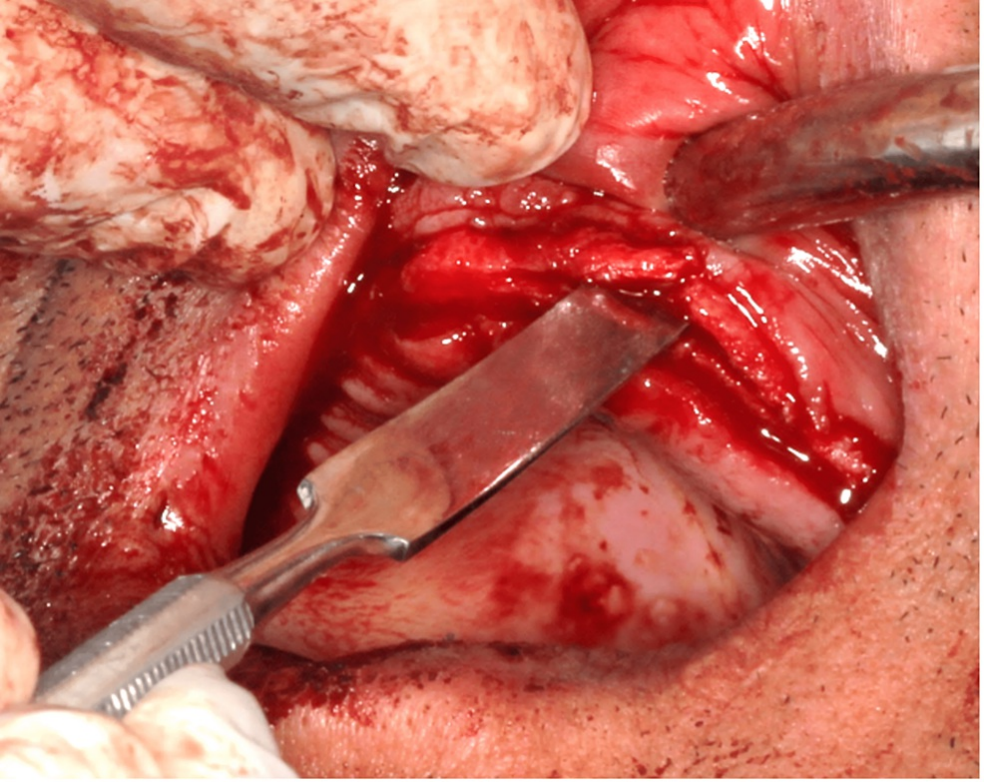
Spatula was inserted allowing further expansion of the bone

Ridge splitting was initiated by gently using a lancet (Bard-Parker stainless steel surgical blades (Aspen Surgical, Caledonia, USA)) (surgical blade number 10) till the ridge was primarily expanded, as shown in Figure 2. Time played a crucial role in the proper bone expansion process, with a minimum of 1 minute required to effectively expand the bone buccally and lingually using the spatula. This duration ensured sufficient and controlled bone expansion, contributing to the success and stability of the bone-splitting procedure. The gradual expansion process also facilitated optimal healing and integration of the augmented bone, promoting the long-term success of the dental implant placement in the expanded site. The inherent properties of the maxillary bone allowed for some slight bony expansion from a crestal approach towards the buccal plate of bone, which was achievable. Subsequently, a round, flat, end scoop spatula (Heathrow Scientific, Vernon Hills, USA) was introduced as an instrument with a broader cross-section, and using it to move the bone buccally and lingually facilitated further bone expansion without causing any fractures. Furthermore, the use of an additional spatula in the same site, where the bone was previously expanded, proved to be very effective in the bone expansion process (Figure 3). 

Starting with the pilot drill, efforts were made to reduce the risk of labial cortical plate fracture. The drills were used to extend each osteotomy site to a depth of 10 mm. Next, osteotomies were prepared and four dental implants (Dentis USA, La Palma, USA), each with a diameter of 3.7 mm and a length of 10 mm, were inserted in the maxillary arch, with two implants on each side. Specifically, two implants were positioned in the maxillary left region, addressing the canine and premolar areas. Additionally, two implants were placed in the maxillary right region, targeting the lateral incisor and premolar areas. The drilling was carried out with precision to achieve the desired depth of 10 mm and ensure stable implant placement. The surgeon applied gentle pressure to ensure optimal stability and primary implant stability. After implant placement, the surgical site was thoroughly irrigated to remove any debris and promote a clean environment for healing. Primary stability was assessed after that, and then closure by interrupted resorbable stitches (4-0 Vicryl sutures Undyed Braided 18" (Ethicon, Raritan, USA)) was carried out.

The patient received a combination of medications to ensure proper healing and manage any discomfort or potential risks. Pain reliever such as ibuprofen (Advil 200 mg) was prescribed (every 4-6 hours) to alleviate post-operative discomfort. Additionally, antibiotics such as amoxicillin (Augmentin 1 gm every 12 hours) were prescribed to prevent or treat any potential infection that could arise after the implant placement. To promote a clean surgical site and minimize infection risk, the patient was advised to use an antibacterial chlorhexidine mouthwash. Furthermore, anti-inflammatory medication, such as corticosteroids like fortacortine, was prescribed to reduce swelling and inflammation at the implant site. Lastly, a mild saltwater rinse was advised to aid in keeping the surgical site clean and support the healing process. It was vital for the patient to follow these instructions carefully regarding medication usage and to report any adverse reactions promptly. The patient was provided with essential oral hygiene practices to promote proper healing and long-term implant success. The patient was advised to maintain good oral hygiene by gently brushing his teeth, including the surgical area, using a soft-bristled toothbrush. To allow for proper healing, he was instructed to avoid brushing directly on the surgical site for the first few days. To support proper healing, the patient was advised to adhere to a soft or liquid diet for a few days after the surgery, steering clear of hard, crunchy, or sticky foods. To further promote successful healing, the patient was advised to refrain from smoking and consuming alcoholic beverages during the healing period, as these habits could interfere with the implant's integration with the surrounding bone. The patient's progress was closely monitored through scheduled follow-up appointments. These appointments allowed the dental team to assess the healing progress, evaluate the integration of the implant with the bone, and ensure that the patient was following proper oral hygiene practices. By diligently adhering to these hygiene instructions and attending regular follow-up visits, the patient took important steps towards ensuring the long-term success and stability of their dental implants, while maintaining overall oral health.

Follow-up and stability assessment

During the follow-up visits for clinical examination, several signs indicated the successful outcome of the dental implant procedure. The implants demonstrated stability without any mobility or movement, indicating secure osseointegration. Radiographic images confirmed osseointegration, revealing the fusion of the implants with the jawbone. The soft tissues around the implants appeared healthy, devoid of inflammation, redness, or swelling. The patient did not report any significant pain or discomfort around the implant site. Follow-up visits showed stable bone levels around the implants, indicating successful long-term stability. The overall health of the peri-implant tissues remained excellent, without signs of recession or inflammation. After five months of osseointegration, the Implants’ stability was measured using a resonance frequency analyzer device (Osstell ISQ (W&H, Bürmoos, Austria)) which revealed successful readings with an average of ISQ 68, 71, 69, and 73 for maxillary left canine, maxillary left premolar, maxillary right lateral incisor and right premolar respectively at the time of surgery. While implant stability at the time of loading was an average of ISQ 69, 68, 65, and 72 for maxillary left canine, maxillary left premolar, maxillary right lateral incisor, and right premolar, respectively. Postoperative CBCT showed an acceptable outcome with good osseointegration.

**Figure 4:**
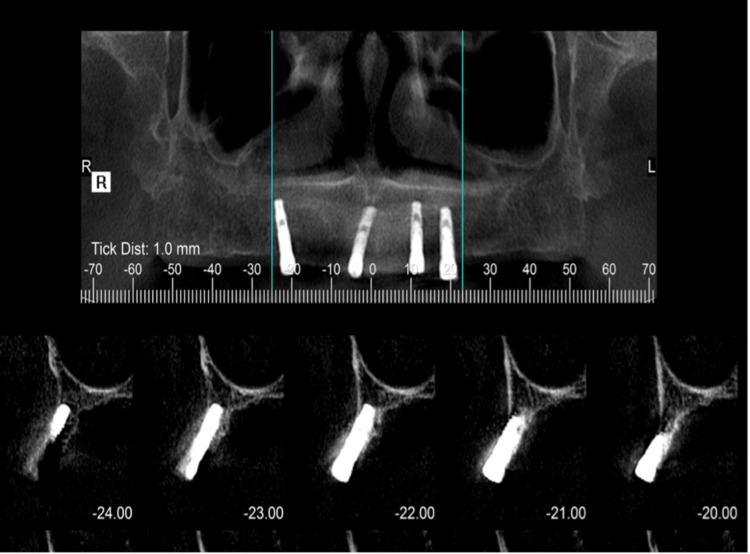
Postoperative CBCT showed an acceptable outcome with good osseointegration CBCT: cone beam computed tomography

Postoperative CBCT with conventional radiographic periapical X-rays showed an acceptable outcome for successful osseointegration. Not in all the cases it is recommended to have a CBCT solely as a tool for the assessment of osteointegration, but in this challenging case, it was considered essential because it offers the advantage of assessing osseointegration. It provides highly accurate and detailed images, allowing for precise evaluation of the implant-bone interface which is vital in determining the success of osseointegration (Figure 4).

Impression-making and implant restorations 

**Figure 5:**
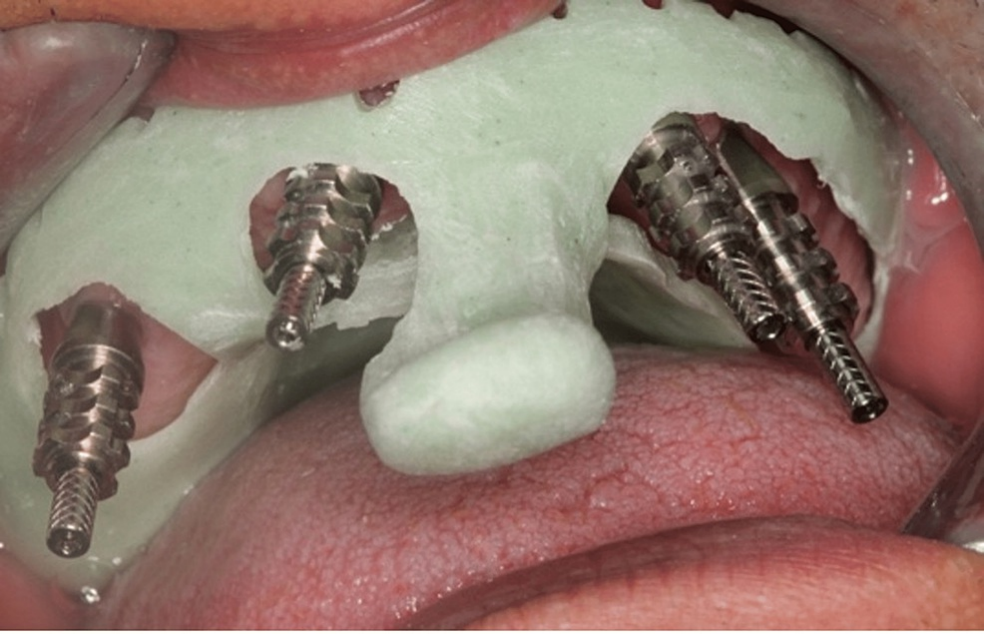
A customized impression tray with openings for the impression copings

**Figure 6:**
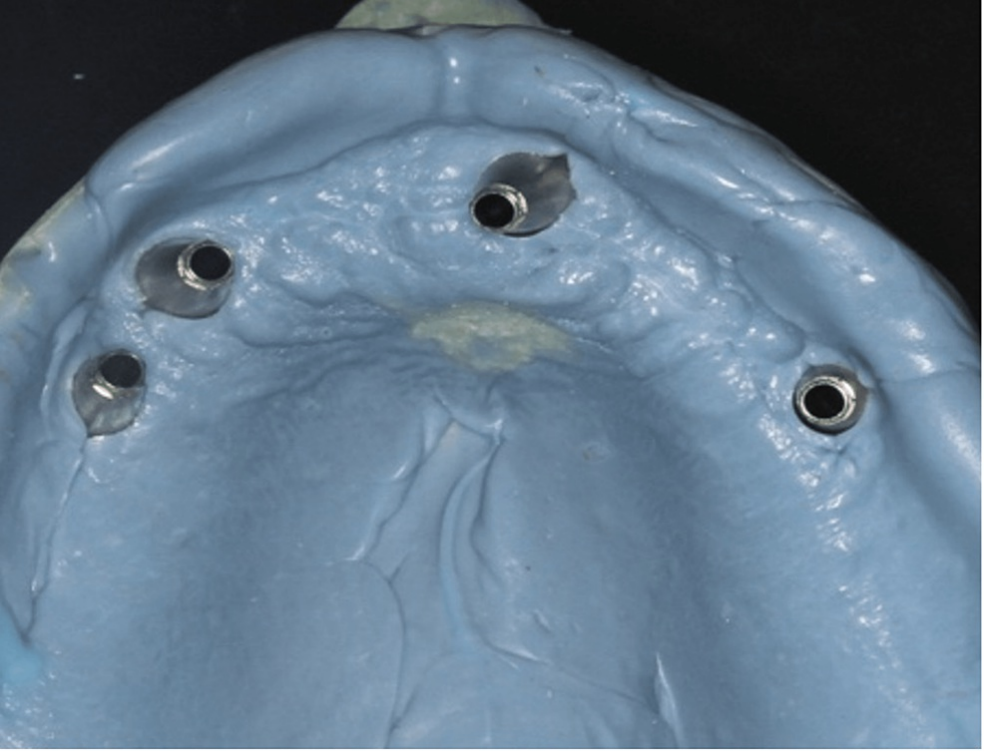
Implant level open tray impression technique

**Figure 7:**
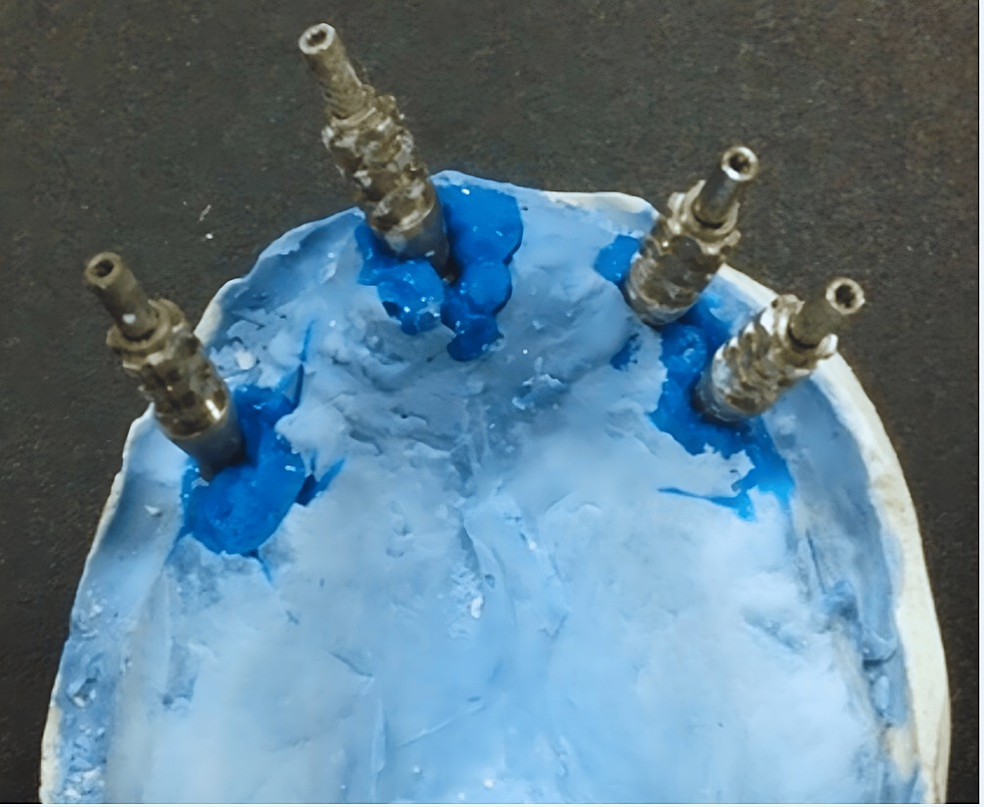
Master cast with impression copings

After the assessment of the stability and osseointegration of the implants impression copings were placed for an implant-level open tray impression technique was taken. The fabrication of a custom tray for the implant impression open tray technique involved several steps. Initially, a preliminary impression was taken using irreversible hydrocolloid alginate (Hydrogum (Zhermack, Marl, Germany)). A stone cast was then prepared from this impression, providing a model of the patient's oral anatomy. A spacer material was applied to create space for the auto-polymerized acrylic resin (Acrostone, London, UK). The custom tray design, which included perforations over the marked implant positions, was developed on the stone cast. The auto-polymerized acrylic resin was mixed and shaped to form the custom tray, covering the designated implant positions. Once the tray was set, it underwent finishing to ensure a comfortable fit with no pressure areas (Figure 5). Then, a final impression was taken using a medium-body polyvinyl siloxane impression material (3M ESPE (3M, St. Paul, USA)) (Figure 6). The impression copings were attached to the implant analog. And finally, a laboratory cast was poured out (Figure 7).

Achieving a prosthetically driven implant placement was challenging because of how deficient the bone was. The angulation of implants was variable; thus, angulated 25° titanium abutments (Dentis USA, La Palma, USA) were considered for the four implants to ensure a common path of insertion and fabricate the final fixed implant prosthesis.

Verification jig

**Figure 8:**
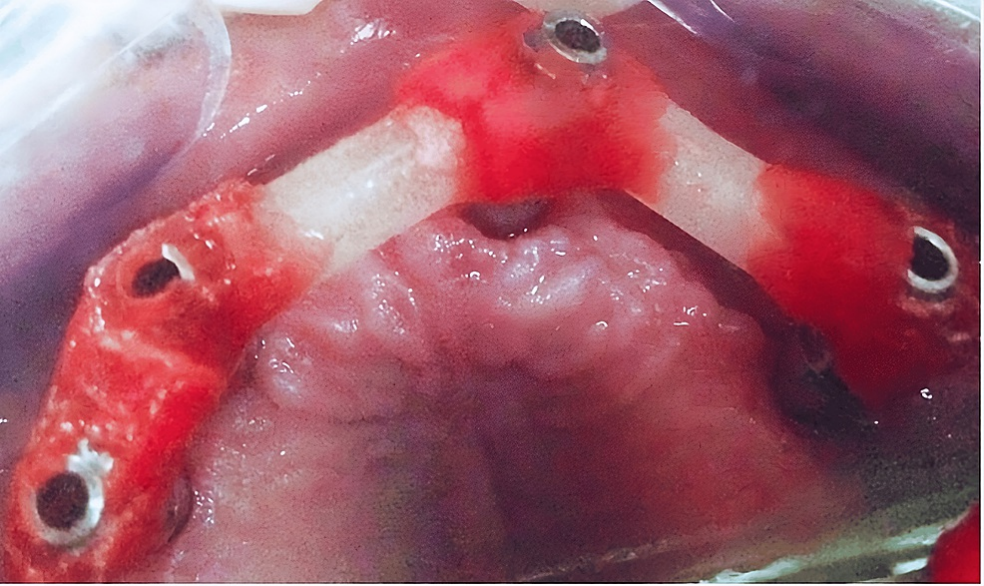
A verification jig to verify the impression intraorally

**Figure 9:**
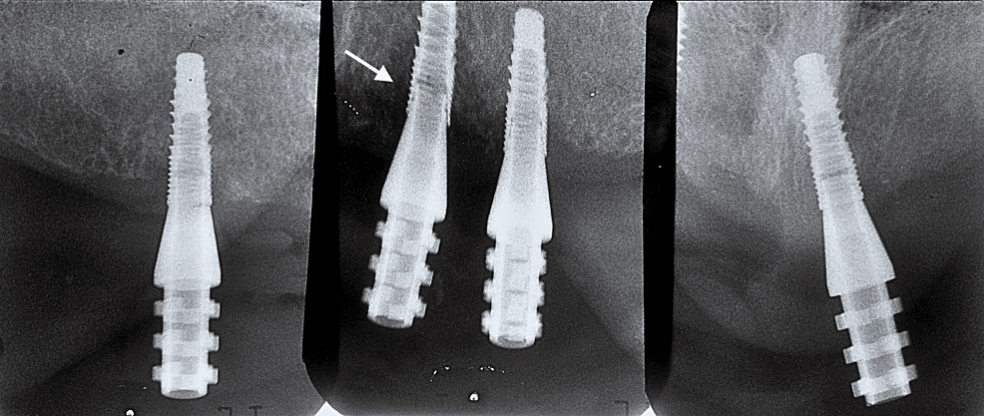
Periapical X-ray confirming that the verification jig was not passively seated

A verification jig (DuraLay Inlay Pattern Resin (Reliance Dental Manufacturing, Alsip, USA)) was made to verify the impression (Figure 8). The verification jig was not passively seated, as confirmed by the X-ray (Figure 9).

**Figure 10:**
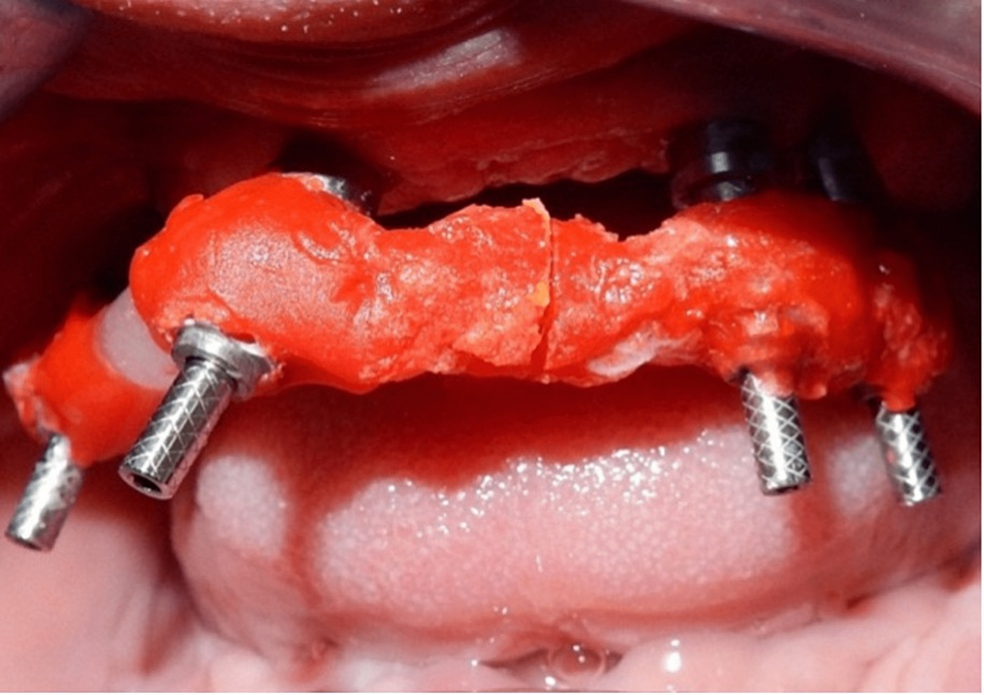
Separation of the jig into three parts

**Figure 11:**
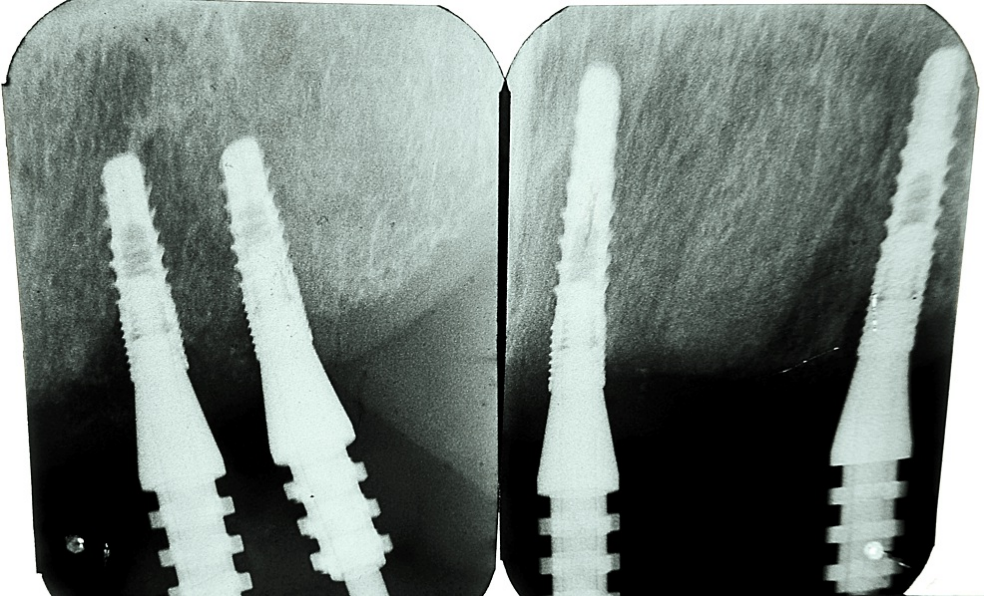
X-ray to confirm passive seating of the verification jig

**Figure 12:**
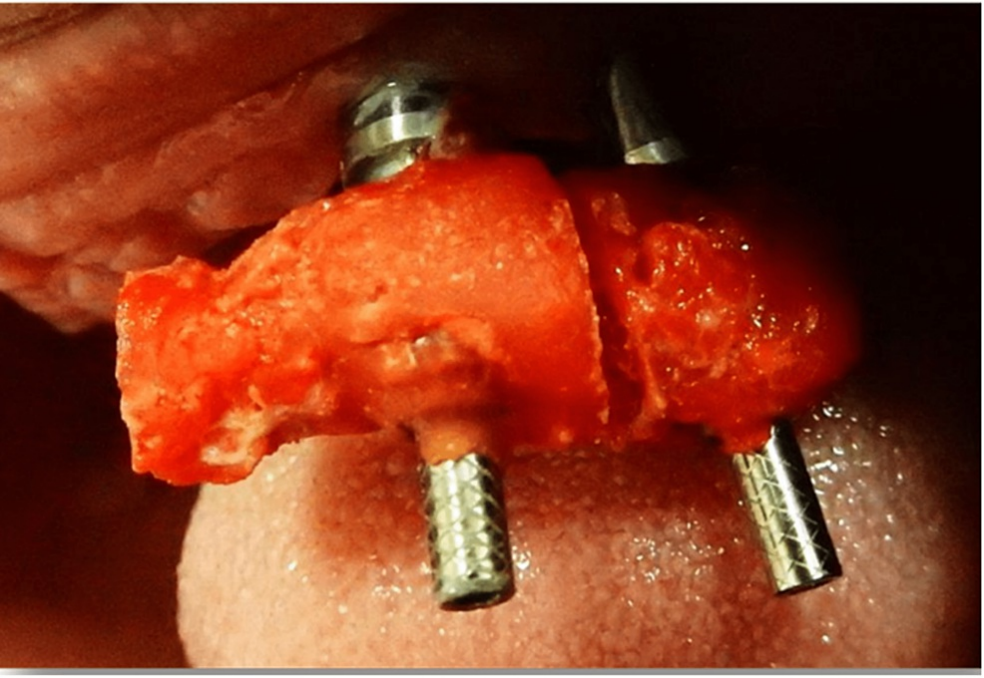
Further sectioning of the verification jig

Further intraoral sectioning was carried out, separating the jig into three parts, followed by splinting intraorally, and seating was also confirmed by an X-ray (Figures 10-12).

Provisional restoration

**Figure 13:**
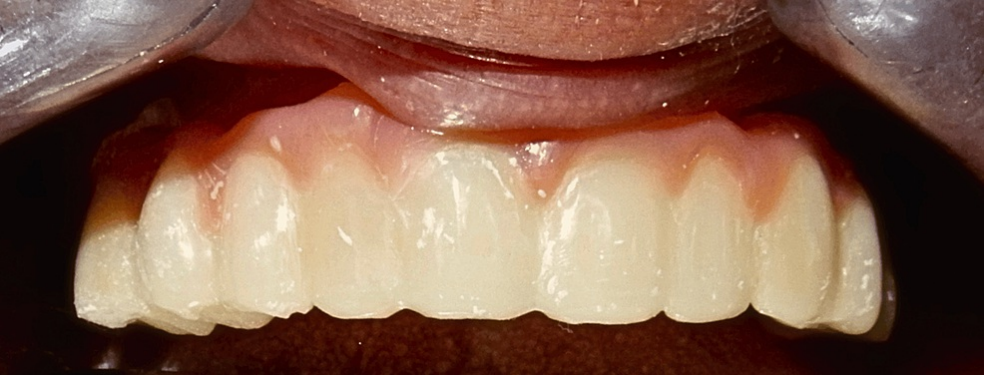
Try-in evaluation of the provisional restoration intraorally

A provisional implant-fixed dental prosthesis, using the Visio-Lign® veneering system (bredent UK, Chesterfield, UK), was designed for the patient. Over the course of four weeks, the prosthesis underwent a series of evaluations and adjustments to ensure optimal function, aesthetics, and speech, all with the goal of meeting the patient's satisfaction before providing the definitive prosthesis (Figure 13).

Metal try-in and fixed prosthesis insertion

**Figure 14:**
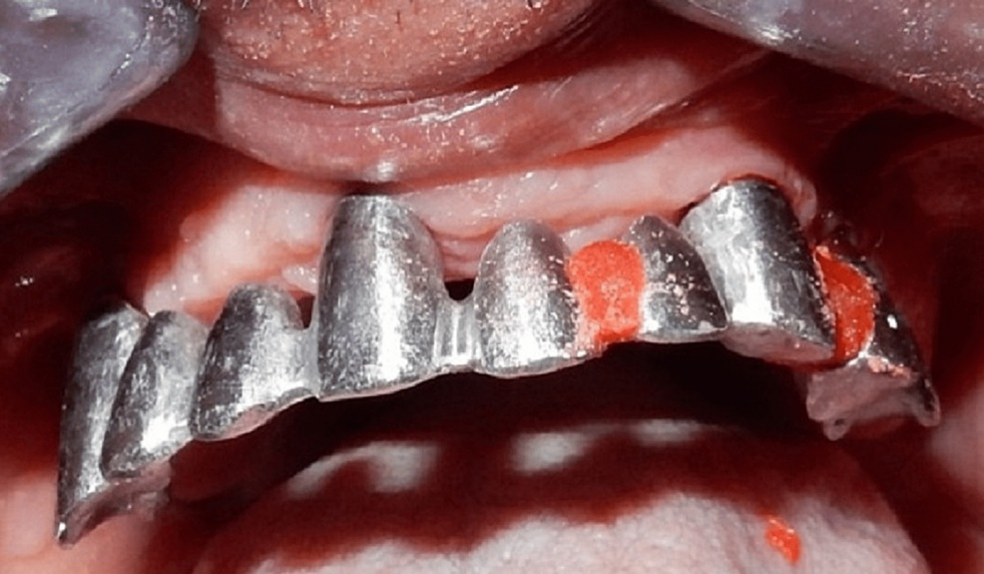
Sectioning and re-splinting of the metal framework due to incomplete seating using acrylic resin

**Figure 15:**
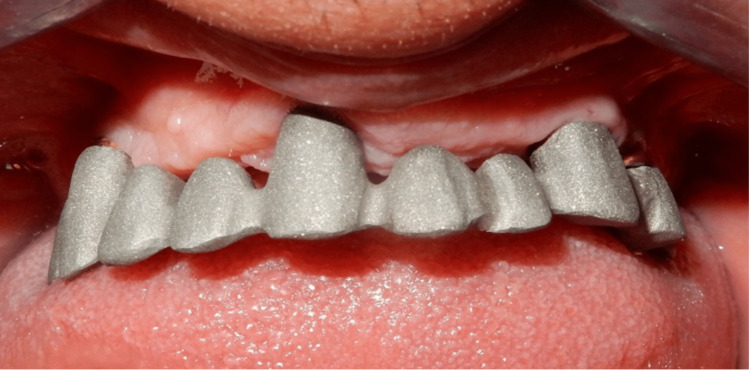
A second try-in for the metal framework after the laboratory adjustments showing passive seating

The metal substructure was tried in four weeks after provisional restoration was delivered, and there was some resistance, so it was sectioned and splinted back with acrylic resin (DuraLay Inlay Pattern Resin) (Figure 14) and sent back to the lab for adjustment. Another try-in was performed and confirmed passive seating without resistance (Figure 15).

**Figure 16:**
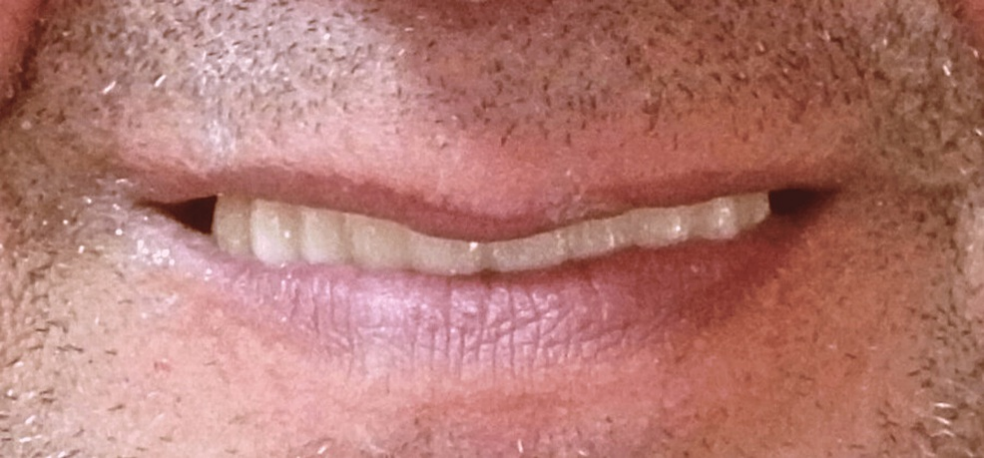
Final PFM restoration checked, adjusted, and cemented PFM: porcelain fused to metal

**Figure 17:**
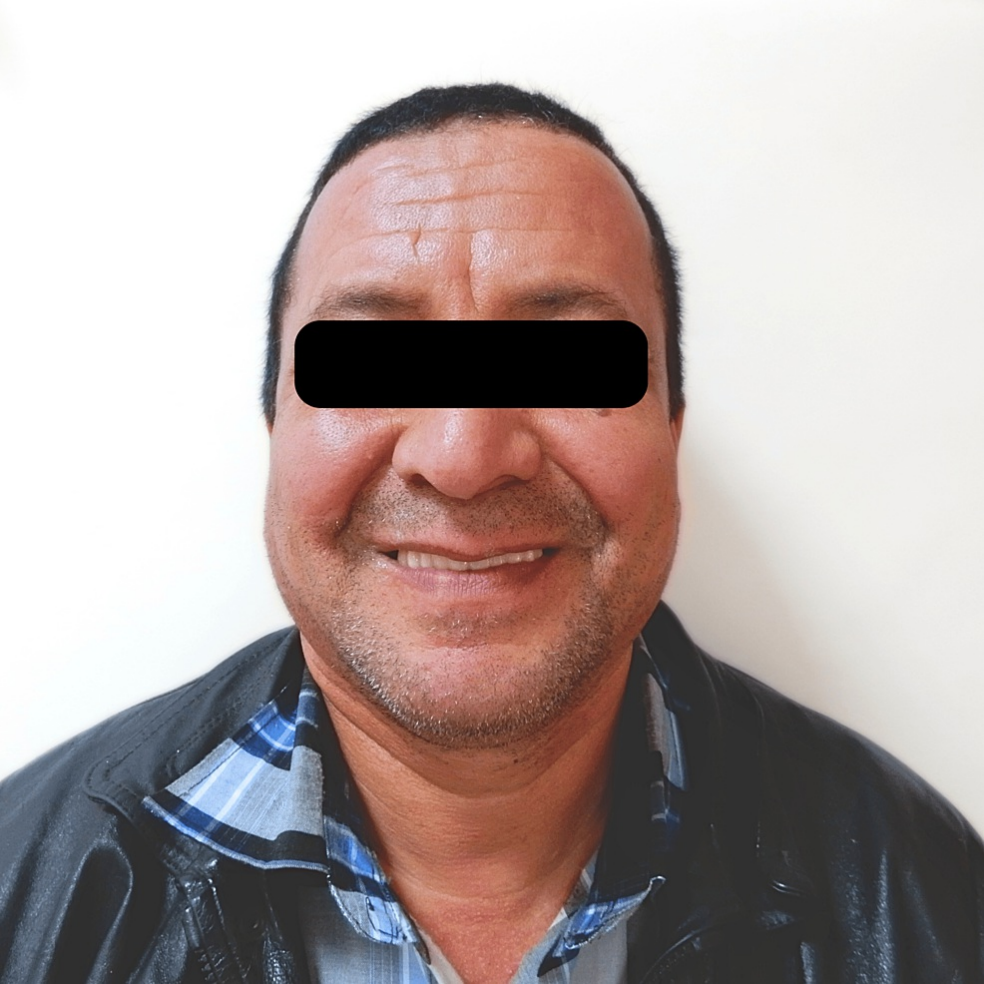
Extraoral picture of the final restoration

The porcelain-fused-to-metal (PFM) restoration was received from the lab for the final insertion. During the delivery, the final implant fixed dental prosthesis was passively seated without misfit problems. The patient was delighted with the aesthetics and the phonetics (Figures 16, 17). Then permanent cementation of the prosthesis (using ZERO-G Bio Implant Cement Kit (Taub Products, Jersey City, USA)) was provided where excessive cement was meticulously removed to avoid biological complications. 

## Discussion

It is widely recognized that an alveolar ridge less than 5 mm necessitates bone augmentation surgery to accommodate an endosseous implant with a good peri‑implant bone of 1.5‑2 mm. Consequences of placing implants in areas that have insufficient ridge width may include fracture of a buccal plate of bone which can lead to implant failure [6]. Therefore, in preparing for a ridge-splitting clinical case, key steps are essential for success. These steps are proper patient assessment, which includes evaluating ridge width and quality through clinical exams and CBCT imaging. Preoperative planning, addressing challenges posed by inadequate ridge width, and considering alternative techniques if buccal bone plate fracture risk is high. Careful instrumentation, controlled osteotomies, and, in some cases, the use of bone grafts or membranes to reinforce the site aim to reduce fracture risk. Informed consent is vital, ensuring patients understand the risks. Post-procedure monitoring is crucial, watching for complications and acting promptly to prevent implant failure [7].

Various treatment options have been described to manage horizontally deficient ridges, including distraction osteogenesis, ridge splitting with bone expansion techniques, increasing width by osteoplasty, using narrow diameter implants, ridge augmentation by autogenous block graft, corticocancellous particulate bone graft, and allograft using GBR membrane, etc. [5].

Bone grafting procedures have shown to be not predictable, and expensive, requiring multiple surgical procedures, and therefore, ridge splitting may be considered as an option. Ridge augmentation is a challenging treatment provided to increase the vertical dimension of the bone, but it requires bone or bone substitute blocks and/or titanium mesh to obtain the desired shape as high complication rates have been reported in the literature. A recent study evaluated the clinical and volumetric outcome after vertical ridge augmentation using Computer-Aided Design/Computer-Aided Manufacturing (CAD/CAM) titanium mesh treated 10 patients for bone augmentation, and the results concluded that 30% of the patients displayed healing complications and 10% of them required a surgical re-entry [8]. A retrospective study evaluating the surgical success of alveolar bone grafting using bone graft before dental implant therapy evaluated 64 bone grafting procedures, and the results concluded that only 49 (71.9%) of the procedures were uneventful without failures or complications. Due to the high complication rates shown in the literature and the need for re-entry surgical procedures, the clinician and patient selected the ridge-splitting approach [9].

Although ridge splitting and bone expansion appear to be technique-sensitive, they have many advantages over different techniques. They allow expansion and simultaneous implant placement without introducing bone graft materials relying solely on the periosteum. The periosteum preservation also helped stabilize the ridge split fracture and reduced the resorption of bony plates and marginal bone [10]. It takes advantage of the inherent quality of flexibility of cancellous bone. Maxillary bone is pliable and can be slowly manipulated to improve quality (compaction and corticalization) and expanded to the desired width [11]. When clinicians allow time to manipulate bone, it will gradually shape to the desired position. It never enables the patient's bone loss, which is commonly inevitable with simple drilling procedures. The effectiveness of this procedure is also dependent on the preservation of labial bone integrity, which happens when the periosteum is intact. Because of its elastic nature, the periosteum serves as a barrier membrane, enables bone expansion and manipulation, and promotes efficient microfracture healing due to an intact blood supply [12].

In comparison to the mandible, the described approach in this research provided an acceptable result in the maxilla bone. Because maxillary bone is less dense, mainly bone of the D2, D3, and D4 types can be molded to the appropriate place [13,14].

A healing period of 5 months was recommended due to the soft nature of the maxillary bone providing the optimum bone-to-implant contact and higher implant stability results [15]. 

Using a series of instruments to separate the ridge in a gentle, patient manner allows for successful manipulation of the bone, decreasing the likelihood of cortical plate fracture. When it occurs, the bone can usually be repositioned, and gentle pressure applied to stabilize it. However, it is believed that the less trauma imposed on the bone during ridge splitting, the faster healing will occur. When the alveolar ridge splitting is performed, the use of a membrane is usually recommended [16].

Although four implants have only been placed in the maxilla, carrying a 10-unit fixed prosthesis might cause excessive loading. However, what was forgiving is that the opposing lower arch was a completely edentulous denture. Moreover, in studies between 5 and 15 years, there is no relationship between the number of implants used to support a complete-arch prosthesis with implant survival rate, prosthesis survival rate, prosthesis complications, or marginal bone loss [17].

The resonance frequency analyzer (RFA) helps to objectively determine implant stability and assess the progress of osseointegration - without jeopardizing the healing process. It is a fast and non-invasive system that provides accurate and objective information to proceed confidently. During the study, the RFA device was employed to measure the resonance frequency of the dental implants at various time points, including during the healing period and after osseointegration. By measuring the implant stability at these different stages, the researchers could track the progress of osseointegration and gather valuable data on the implant's stability over time. The data collected from the RFA device was analyzed to evaluate the success of osseointegration and identify any potential issues or complications that could arise during the healing process. The stability values obtained from the RFA measurements were compared with established benchmarks and previous studies to assess the implant's performance and integration with the surrounding bone. Based on the RFA data, the healthcare team could make informed decisions regarding the patient's treatment plan. If the implant stability values fell within the expected range, it indicated successful osseointegration, and the healthcare team could proceed confidently with the next steps in the treatment process. On the other hand, if the RFA measurements showed lower than anticipated stability values, it might have signaled potential concerns with osseointegration. In such cases, the health care team could intervene promptly, reevaluate the healing progress, and consider additional measures to support successful osseointegration. This proactive approach allowed the team to address any challenges promptly, potentially reducing the risk of implant failure or complications [18].

Despite its disadvantages, the use of a cement-retained full-mouth prosthesis was considered the most viable treatment option in our complex case. Extensive edentulous areas and challenging implant angulations necessitated a cement-retained approach because it provides a more passive fit and precise occlusion, ensuring long-term stability and patient comfort. Additionally, cost-effectiveness was a priority, and cement-retained prostheses offered a satisfactory restoration without compromising quality. The simplicity of the cementation process intraorally and patient preference further justified the decision to opt for cement-retained prostheses in this specific case. Moreover, the meticulous removal of the excess cement was a prime concern to avoid any biological complications [19].

Currently, clinicians can make the final implant impression with novel technology with intra-oral scanners or with traditional methods with polyvinyl siloxane material, and this case was done with traditional techniques because of its proven accuracy. A recent study comparing the accuracy between digital and conventional implant impressions did two- and three-dimensional evaluations. The study assessed intraoral optical scans and conventional impressions with resin models and stone casts, and the dimensions were measured five times. The results concluded that for long-span the precision of digital impressions was significantly inferior to the traditional impressions [20]. Moreover, a comparative in vitro study evaluated the accuracy of stone casts from conventional implant impressions versus casts generated with digital implant impressions. They did the study on a stone cast with Kennedy class II edentulism. Conventional impressions were made with the open tray technique and digital impressions were made with two different scanners (white light and active wavefront). The results concluded that conventional stone casts were the most accurate (54.49 μm), followed by white light scanner (108.09 μm) and active wavefront scanner (120.39 μm). The study concluded that printed casts from digital impressions had significantly inferior accuracy than traditional stone casts fabricated from impressions with polyether material [21]. Due to the evidence showing that traditional techniques for implant impression and conventional casts are more accurate than novel technologies, this patient was treated with traditional methods. 

An accurate three-dimensional reproduction of the intraoral position of the implants through the impression phase is necessary. Clinically, additional factors, such as the number, angulation, depth of implants, and impression materials, may affect the accuracy of implant impressions [21]. Therefore, a verification jig is mandatory to improve accuracy for an optimal passive prosthetic fit. Its use has been suggested to eliminate or minimize misfits when implants are joined [22].

The veneering system for fixed implant prostheses comprises multi-layer veneers developed from natural teeth and a bonding system in perfectly matched shades. It is characterized by high shade stability and resistance to plaque and abrasion. The veneering of implant-supported restorations with a high-performance polymer represents today's method of choice in implant prosthetics. The cushioning properties against the chewing pressure of these materials in comparison to ceramic veneer materials should be particularly highlighted [23].

The literature already has some case reports with ridge-splitting techniques. A recent report shows a mandibular alveolar ridge split with simultaneous implant placement for a 47-year-old patient in the lower left jaw region. The report shows how the ridge was expanded with hand osteotomes followed by two 3.75 mm x 11.5 mm implants on sites for the first and second molar [24]. Another publication is a report of three cases of partially edentulism patients treated with alveolar ridge management with a modified ridge technique in which for the first patient a maxillary right molar was extracted simultaneously with ridge splitting and implant placement after 4 months. In the second case, the mandibular left first molar was extracted simultaneously with ridge splitting and immediate implant placement on the first premolar and first premolar sites. The last report included ridge splitting and simultaneous implant placement for sites upper right first molar and second premolar [25]. Our case report represents a more challenging clinical situation because it is in the esthetic zone and the patient is fully edentulous. 

The limitations of this case report could be related to the demonstration of a single case, so further reports should be case series to validate the treatment in different clinical scenarios. Another limitation could be the use of only one implant brand, so it could be interesting to see the performance of similar reports with different brand systems. Lastly, the procedures were performed by experienced clinicians in the field, and the results may be different for novice clinicians, so randomized trials comparing the training and skills of the clinicians may be interesting.

## Conclusions

The ridge-splitting technique offers the great advantage of placing a dental implant at the same surgical appointment in ≥3 mm of bone width. In this case, the patient's alveolar ridge experienced a net gain of more than 3 mm, enabling the placement of 3.7 mm-wide implants. This modified ridge-splitting technique is a simple and short procedure with satisfactory results and minimal morbidity. The approach overcomes the disadvantages of bone grafting and has a low cost.

## References

[1] Shiezadeh F, Arab HR, Khoshkam V, Moeintaghavi A, Forouzanfar A, Khodadadifard L (2023). Comparison of guided bone regeneration with periosteal pocket flap technique versus autogenous bone block graft for horizontal bone augmentation: a clinical trial study. Int J Periodontics Restorative Dent.

[2] Assem NZ, Pazmiño VF, Rodas MA (2023). Bone substitutes graft for regeneration of the anterior maxillary alveolar process: a systematic review. J Oral Implantol.

[3] Herford AS (2005). Distraction osteogensis: a surgical option for restoring missing tissue in the anterior esthetic zone. J Calif Dent Assoc.

[4] Chopra M, Kumar M, Vermani M, Swarup N (2021). Cast-based guided implant placement and prosthetic rehabilitation of a single edentulous space: case report and systematic analysis of surgical guides used in implant dentistry. J Long Term Eff Med Implants.

[5] Manekar VS, Shenoi SR, Manekar S, Jhon J (2022). Alveolar ridge split and expansion with simultaneous implant placement in mandibular posterior sites using motorized ridge expanders - modified treatment protocol. Natl J Maxillofac Surg.

[6] Khairnar MS, Khairnar D, Bakshi K (2014). Modified ridge splitting and bone expansion osteotomy for placement of dental implant in esthetic zone. Contemp Clin Dent.

[7] Arora V, Kumar D (2015). Alveolar ridge split technique for implant placement. Med J Armed Forces India.

[8] Cucchi A, Bianchi A, Calamai P, Rinaldi L, Mangano F, Vignudelli E, Corinaldesi G (2020). Clinical and volumetric outcomes after vertical ridge augmentation using computer-aided-design/computer-aided manufacturing (CAD/CAM) customized titanium meshes: a pilot study. BMC Oral Health.

[9] Schwartz-Arad D, Levin L, Sigal L (2005). Surgical success of intraoral autogenous block onlay bone grafting for alveolar ridge augmentation. Implant Dent.

[10] Deng C, Yi Z, Xiong C, Man Y, Qu Y (2022). Using the intact periosteum for horizontal bone augmentation of peri-implant defects: a retrospective cohort study. Br J Oral Maxillofac Surg.

[11] Jaffin RA, Berman CL (1991). The excessive loss of Branemark fixtures in type IV bone: a 5-year analysis. J Periodontol.

[12] Summers RB (1994). A new concept in maxillary implant surgery: the osteotome technique. Compendium.

[13] Misch CE (1989). Bone classification, training keys to implant success. Dent Today.

[14] Seong WJ, Kim UK, Swift JQ, Heo YC, Hodges JS, Ko CC (2009). Elastic properties and apparent density of human edentulous maxilla and mandible. Int J Oral Maxillofac Surg.

[15] Gehrke SA, Tavares da Silva Neto U (2014). Does the time of osseointegration in the maxilla and mandible differ?. J Craniofac Surg.

[16] Moro A, Gasparini G, Foresta E (2017). Alveolar ridge split technique using piezosurgery with specially designed tips. Biomed Res Int.

[17] de Luna Gomes JM, Lemos CA, Santiago Junior JF, de Moraes SL, Goiato MC, Pellizzer EP (2019). Optimal number of implants for complete-arch implant-supported prostheses with a follow-up of at least 5 years: a systematic review and meta-analysis. J Prosthet Dent.

[18] Manzano-Moreno FJ, Herrera-Briones FJ, Bassam T, Vallecillo-Capilla MF, Reyes-Botella C (2015). Factors affecting dental implant stability measured using the ostell mentor device: a systematic review. Implant Dent.

[19] Hamed MT, Abdullah Mously H, Khalid Alamoudi S, Hossam Hashem AB, Hussein Naguib G (2020). A systematic review of screw versus cement-retained fixed implant supported reconstructions. Clin Cosmet Investig Dent.

[20] Fu Y, Yin C, Li S, Li D, Mo A (2022). A full digital workflow to prefabricate an implant-supported interim restoration: case report and a novel technique. Int J Implant Dent.

[21] Kim HS, Lee JH, Lee SY (2022). Accuracy of impression techniques for dental implants placed in five different orientations. Int J Oral Maxillofac Implants.

[22] De La Cruz JE, Funkenbusch PD, Ercoli C, Moss ME, Graser GN, Tallents RH (2002). Verification jig for implant-supported prostheses: a comparison of standard impressions with verification jigs made of different materials. J Prosthet Dent.

[23] Diken Turksayar AA, Donmez MB (2023). Stress behavior of an anterior single implant restored with high-performance polymer abutments under immediate and delayed loading: A 3D FEA study. J Prosthodont.

[24] Pandey KP, Kherdekar RS, Advani H, Dixit S, Dixit A (2022). Mandibular alveolar ridge split with simultaneous implant placement: a case report. Cureus.

[25] Kim HY, Park JH, Kim JW, Kim SJ (2023). Narrow alveolar ridge management with modified ridge splitting technique: a report of 3 cases. Case Rep Dent.

